# The effect of elevated physiological temperatures on bacterial survival and antibiotic susceptibility in an *in vitro* infection prevention model

**DOI:** 10.3389/fmicb.2026.1830056

**Published:** 2026-05-29

**Authors:** Robert Kamphof, Marielle Verheul, Rob G. H. H. Nelissen, Mark G. J. de Boer, Bart G. C. W. Pijls

**Affiliations:** 1 Department of Orthopaedics, Leiden University Medical Center, Leiden, Netherlands; 2 Department of Clinical Epidemiology, Leiden University Center for Infectious Diseases (LUCID), Leiden University Medical Center, Leiden, Netherlands

**Keywords:** heat, hyperthermia, hypothermia, infection prevention, normothermia, implants, arthroplasty

## Abstract

**Aims:**

Prosthetic joint infection (PJI) is a devastating adverse outcome of total joint arthroplasty surgery, and is often accompanied by treatment failure. Due to a rise in the number of orthopaedic implants and global antimicrobial resistance, non-antibiotic strategies for preventing and treating orthopaedic infections are needed. This study investigates the effect of temperature in the physiological range on bacterial killing, bacterial adhesion and the antibiotic susceptibility of bacteria.

**Methods:**

Planktonic phase clinical isolates of methicillin-sensitive *Staphylococcus aureus*, methicillin-sensitive *Staphylococcus epidermidis* and *Escherichia coli* at 103 colony forming units (CFU)/mL were exposed for 24 h to 32 °C, 37 °C, 40 °C, and 42 °C with and without cefazolin or gentamicin in the subtherapeutic to therapeutic range. Thereafter, the bacterial load in the planktonic and adhered fractions was enumerated in CFU/mL. Dose–response curves were fitted into the data to find the effect of temperature on the antibiotic dose needed for bacterial eradication.

**Results:**

Increasing the temperature from 32 to 42 °C reduced the minimum concentration required to kill *Staphylococcus aureus* from 0.5 to 0.05 μg/mL for both gentamicin and cefazolin. Almost full eradication of *Staphylococcus aureus* was achieved at 42 °C without antibiotics. *Staphylococcus epidermidis* demonstrated enhanced susceptibility to cefazolin at increased temperatures, with a 6-log reduction in CFU/mL upon exposure to 0.1 μg/mL cefazolin at 42 °C compared to lower temperatures. Notably, antibiotic efficacy at 32 °C was impaired compared to 37 °C. *Escherichia coli* did not exhibit significant changes in CFU/mL at any temperature.

**Conclusion:**

These *in vitro* data strongly indicates that elevated temperatures within the physiological range effectively reduce the bacterial load and increase antibiotic susceptibility of the most common Gram-positive pathogens found in PJI. Conversely, hypothermic conditions reduced the bacterial susceptibility to antibiotics. Since the risks involved within the tested temperature range are minimal, applying elevated temperatures to prevent orthopaedic infections may have clinical benefits.

## 1 Introduction

Prosthetic joint infection (PJI) is a major reason for failure of joint replacement (Tande and Patel, 2014). PJI is usually caused by gram-positive bacteria such as *Staphylococcus aureus* (*S. aureus*) and *Staphylococcus epidermidis* (*S. epidermidis*), but can also be induced by other microorganisms, such as gram-negative *Escherichia coli* (*E. coli*), or be polymicrobial (Tande and Patel, 2014). Treatment of PJI is complicated by bacterial biofilm formation on the implant surface (Hall and Mah, 2017; Staats et al., 2021). These biofilm structures consist of bacteria embedded within self-produced extracellular polymeric substances, reducing the efficacy of the host immune system to eradicate the infection. Bacteria within biofilms are also highly tolerant against many antibiotics that are currently used to treat PJI (Hall and Mah, 2017). Because of the challenges involved in treating established biofilms, preventing their formation is key.

Current clinical practice commonly employs cefazolin as antimicrobial prophylaxis for prosthetic joint surgery (Lutro et al., 2025). In addition, gentamicin is often loaded into orthopaedic bone cement to prevent the infection of cemented implants (Van De Belt et al., 2000; Neut et al., 2010). However, these approaches do not prevent PJI in all cases. This might be due to surgical and patient factors, but notably, the global rise of antimicrobial resistance (AMR) is reducing the efficacy of many antibiotics used for surgical prophylaxis (Murray et al., 2022; Agtmaal et al., 2025). Hence, new strategies for reducing the incidence of PJI that do not solely rely on antibiotics are urgently needed, such as the use of elevated temperatures to prevent infections.

Hyperthermia (in the form of fever) is part of the natural immune response, and can induce bacterial cell damage and enhance bacterial susceptibility to antibiotics (Evans et al., 2015). At the same time, elevated temperatures can stimulate effector functions of host immune cells. Earlier studies showed that low temperatures generally require higher antibiotic doses to be effective (Hinks et al., 1977; Mackowiak et al., 1982). However, these studies only addressed parts of the physiological temperature range, and did not present quantitative data on bacterial killing. To our knowledge, studies investigating the effect of temperature alone on bacterial killing are missing. Therefore, the aim of this study was to investigate the effects of temperatures within the whole physiological range, and the combination of temperature with antibiotics to prevent implant-associated infection caused by *S. aureus*, *S. epidermidis*, and *E. coli in vitro*. We hypothesised that elevated temperatures mimicking natural fever conditions induce bacterial killing and hamper bacterial adhesion (Evans et al., 2015). For this, bacterial survival and adherence across the whole physiological range, ranging from 32 °C (hypothermia) to 42 °C (mild hyperthermia) on bacterial load is determined.

## 2 Methods

### 2.1 Bacterial isolates

This study used clinical isolates of methicillin-sensitive *Staphylococcus aureus* (MSSA, from a patient with PJI, coded as LUH15393), methicillin-sensitive *Staphylococcus epidermidis* (MSSE, from a patient with PJI, coded as LUH15408), and *Escherichia coli* (*E. coli*, from a patient with urinary tract infection, coded as LUH15174) which were stored in glycerol stocks at −70 °C. All bacteria strains used for this study were susceptible to cefazolin and gentamicin.

### 2.2 Bacterial culture

Bacteria from frozen glycerol stocks were spread on trypticase soy agar plate with 5% sheep blood (Biomérieux, France) and incubated overnight at 37 °C. Subsequently, colonies from this plate were cultured to mid-logarithmic growth phase in tryptone soy broth (TSB, CM0129, Thermo Fisher, USA) for 2.5 h (*S. aureus*, *E. coli*) or 3.5 h (*S. epidermidis*) at 37 °C and 200 rpm. Bacteria were then centrifuged (2068 RFC, 10 min) and washed with phosphate-buffered saline (PBS, pH 7.4, Sigma-Aldrich, USA). Based on optical density (OD_600nm_), bacteria were diluted to 2 × 10^3^ colony forming units (CFU)/mL in brain-heart infusion (BHI, CM1135, Thermo Fisher, USA) broth, which is considered a suitable starting inoculum for an infection prevention model (Tande and Patel, 2014; Sjollema et al., 2018). This starting concentration was controlled by microbiological evaluation of the bacterial load in CFU/mL.

### 2.3 Antibiotics

Cefazolin (solid powder, Mylan, U.K.), was used at concentrations of 0.05, 0.1, 0.25 and 0.5 μg/mL for *S. aureus* and *S. epidermidis* and 0.1, 0.5 and 1 μg/mL for *E. coli*. Gentamicin stock solution 50mg/L in Milli-Q water (Merck, USA) was used at concentrations of 0.01, 0.05, 0.1, 0.5, 1 and 2 μg/mL. Antibiotics were pre-diluted in Milli-Q water and thereafter in BHI. Cefazolin and gentamicin were stored at 20 °C and 4 °C, respectively.

### 2.4 Infection prevention assay utilizing antibiotics, hypothermia, and hyperthermia

The bacterial suspension and antibiotics in BHI were mixed (50 μL each) in flat-bottom 96-wells polystyrene plates (Greiner Bio One, Austria). BHI was used as antibiotic-free condition and medium controls were included to control for any possible contamination. Plates were sealed using a breathable sealing film (391-1262, VWR, USA) and cultured under static conditions for 24 h at various temperatures: (i) 32 °C, representing low physiological temperatures as found in distal joints and joints during surgery, (ii) 37 °C, representing normal core body temperature, (iii) 40 °C, representing natural fever conditions, (iv) 42 °C, representing extreme physiological temperatures. For gentamicin, bacteria were only exposed to 32, 37 and 42 °C. The upper temperature limit of 42 °C was chosen, since human cells are generally able to tolerate temperatures up 43 °C before suffering acute damage (Van Rhoon et al., 2013). Temperatures as low as 32 °C can be measured in distal joints, especially during surgery (Gavish et al., 2023; De Marziani et al., 2025).

After 24 h, the culture media was carefully removed from the wells to enumerate the planktonic bacterial load. Further, the wells were carefully rinsed two times with PBS to remove residual non-adhered bacteria. PBS was added to each well and the plate was sealed using an aluminium sealing film (Corning, USA). Subsequently, adhered bacteria were harvested from the wells by sonication (40 kHz, 10 min) followed by resuspension. Both the planktonic and adhered fractions were 10× serially diluted in PBS and plated on Mueller-Hinton agar (CM0337, Thermo Fisher, USA) plates. The bacterial load was enumerated in CFU/mL after overnight incubation at 37 °C. Based on the plated volume of 20 uL, the limit of detection was 50 CFU/mL.

### 2.5 Data analysis

All experiments were performed at least three times in triplicate. The results are reported as mean log(CFU/mL) with corresponding 95% confidence interval, which were calculated using Rstudio (version 2025.05.0). The antibiotic concentration required to eradicate all bacteria was reported if relevant.

Dose response curves were fitted to the data using the DRC package in Rstudio (Ritz et al., 2015). Data were stratified by type of bacteria (species, planktonic/adhered) and temperature. For each study group, the log(CFU/mL) was fitted against the antibiotic concentration using a log-logistic curve with fixed lower limit of 0. The antibiotic dose needed to reduce the log(CFU/mL) by half (ED50) was reported. The code used to generate the results and figures is available in the Supplementary information.

## 3 Results

**Figure 1:**
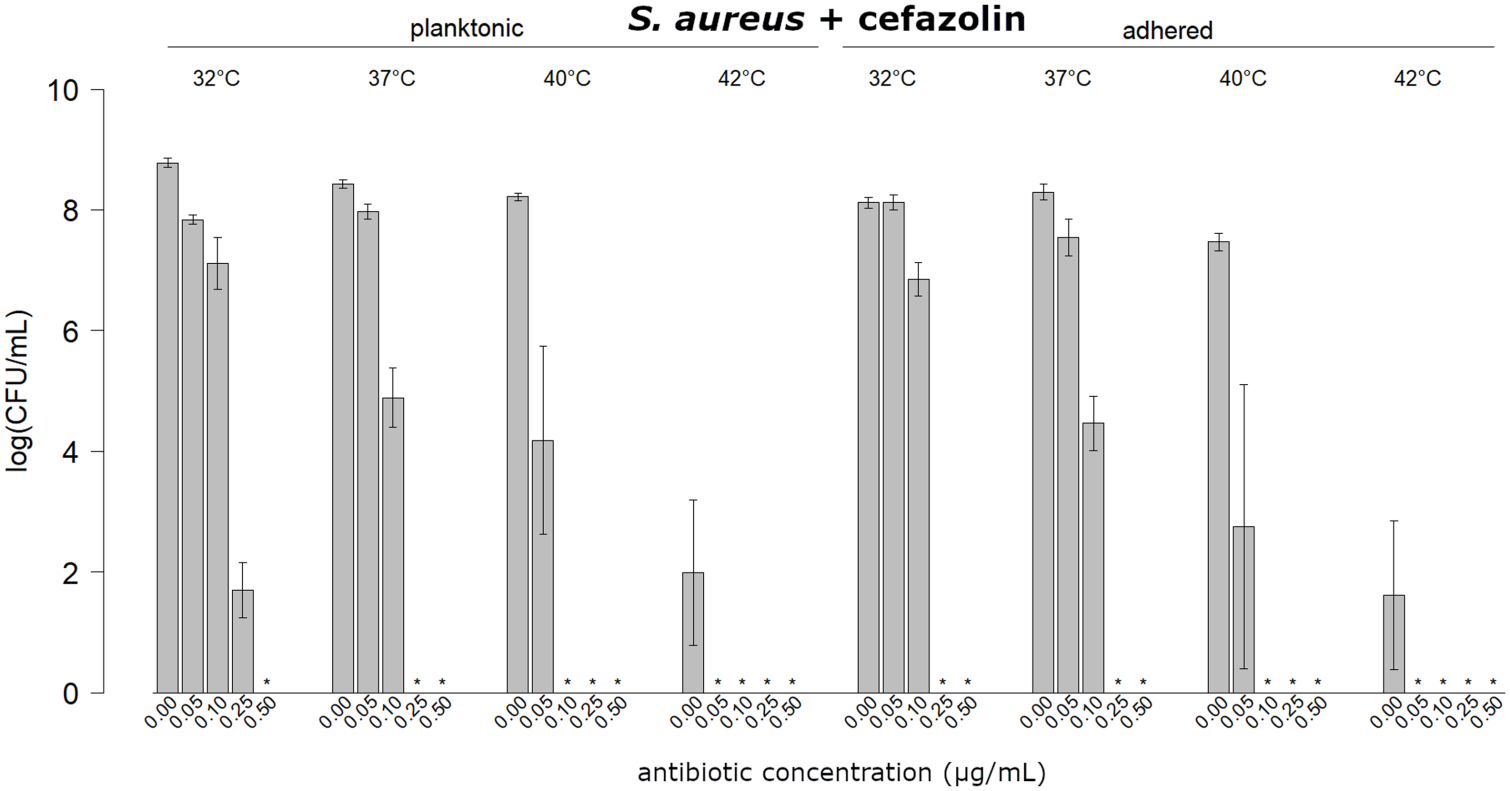
Remaining bacteria titres for *S. aureus* with cefazolin after 24 h of culture at different antibiotic concentrations and ambient temperatures. Concentrations are in μg/mL. Results are reported as log(CFU/mL) ± 95% CI.

**Figure 2:**
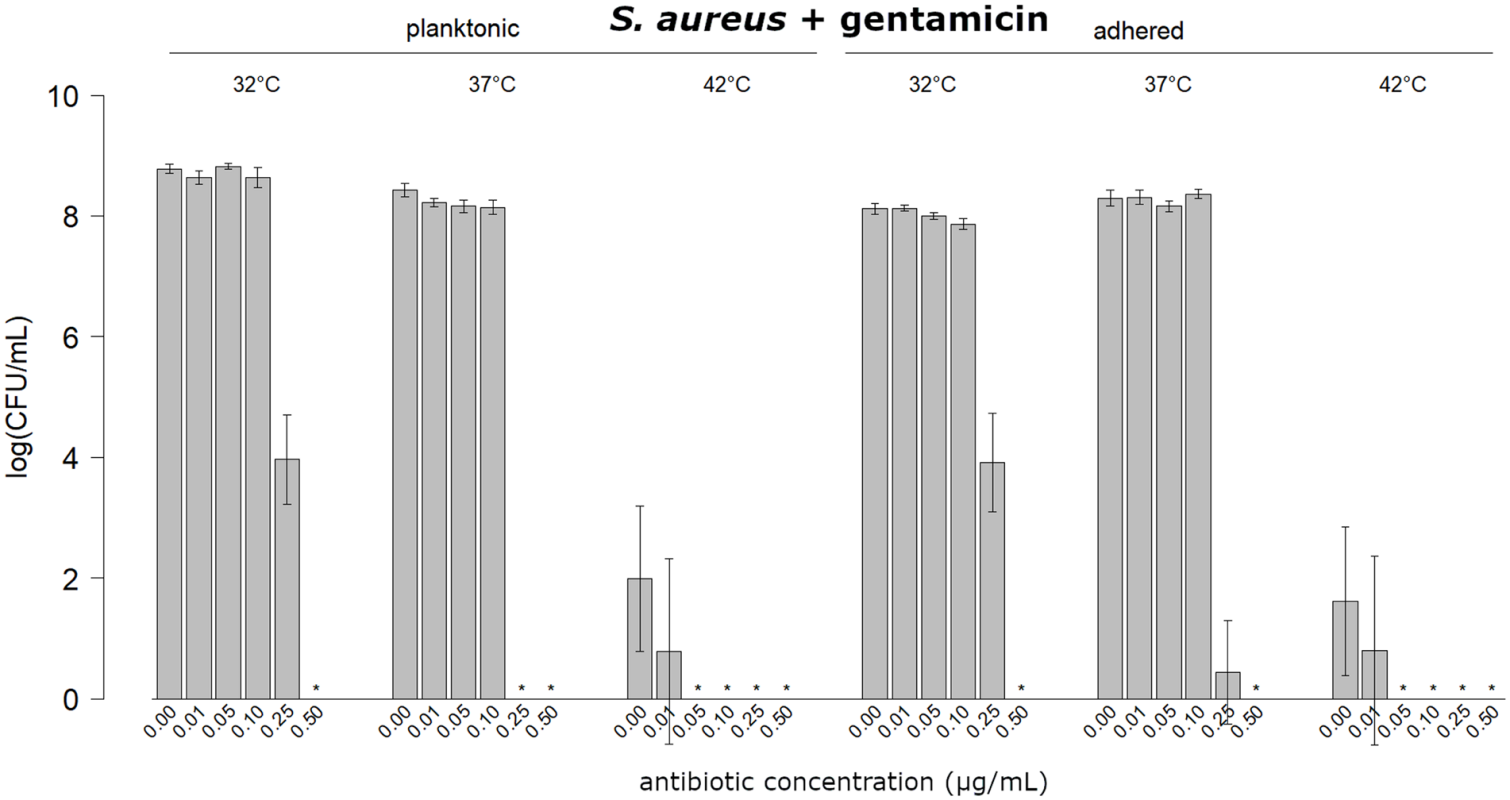
Remaining bacteria titres for *S. aureus* after 24 h of culture at different antibiotic concentrations and ambient temperatures. Concentrations are in μg/mL. Results are reported as log(CFU/mL) ± 95% CI.

**Figure 3:**
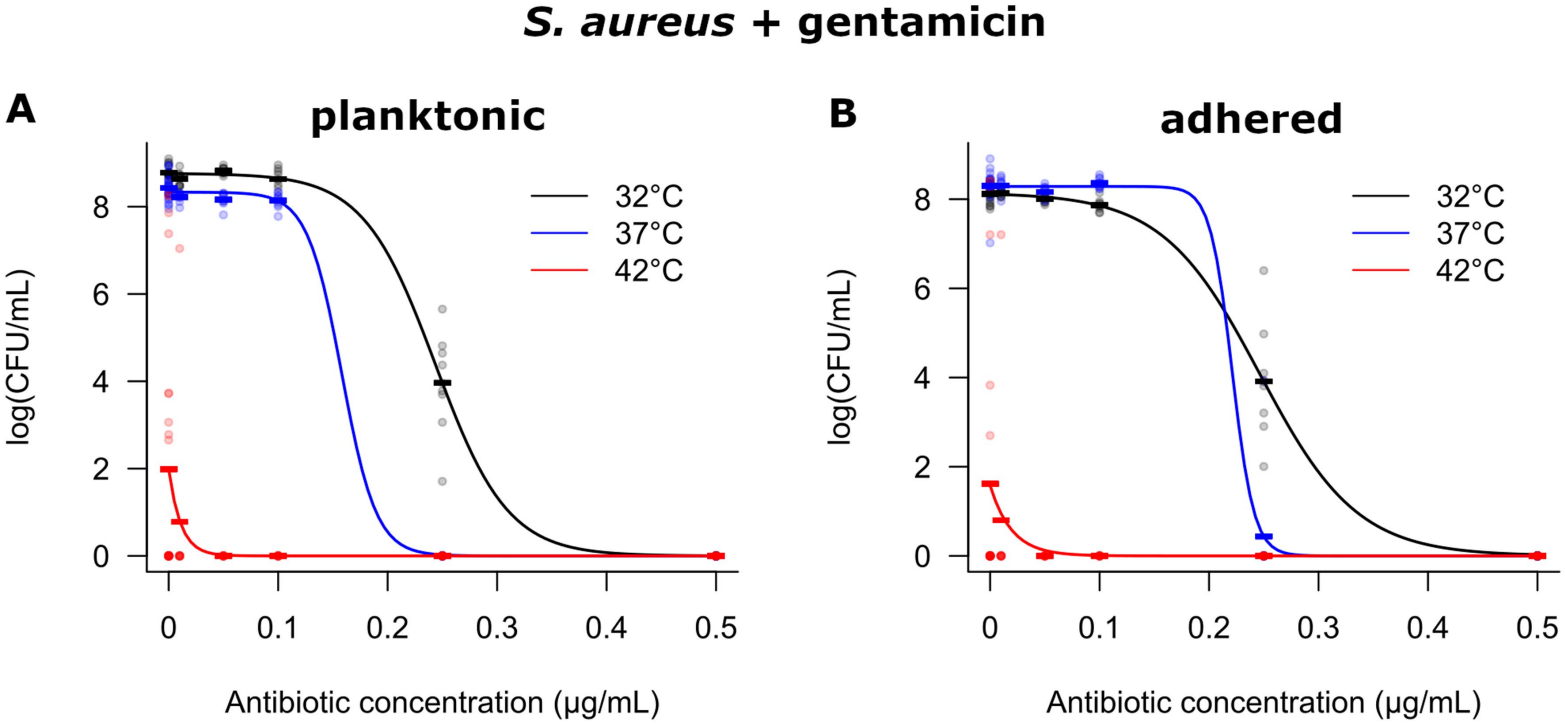
Calculated log-logistic curves for the planktonic **(A)** and adherent **(B)** fractions of *S. aureus* with gentamicin.

**Figure 4:**
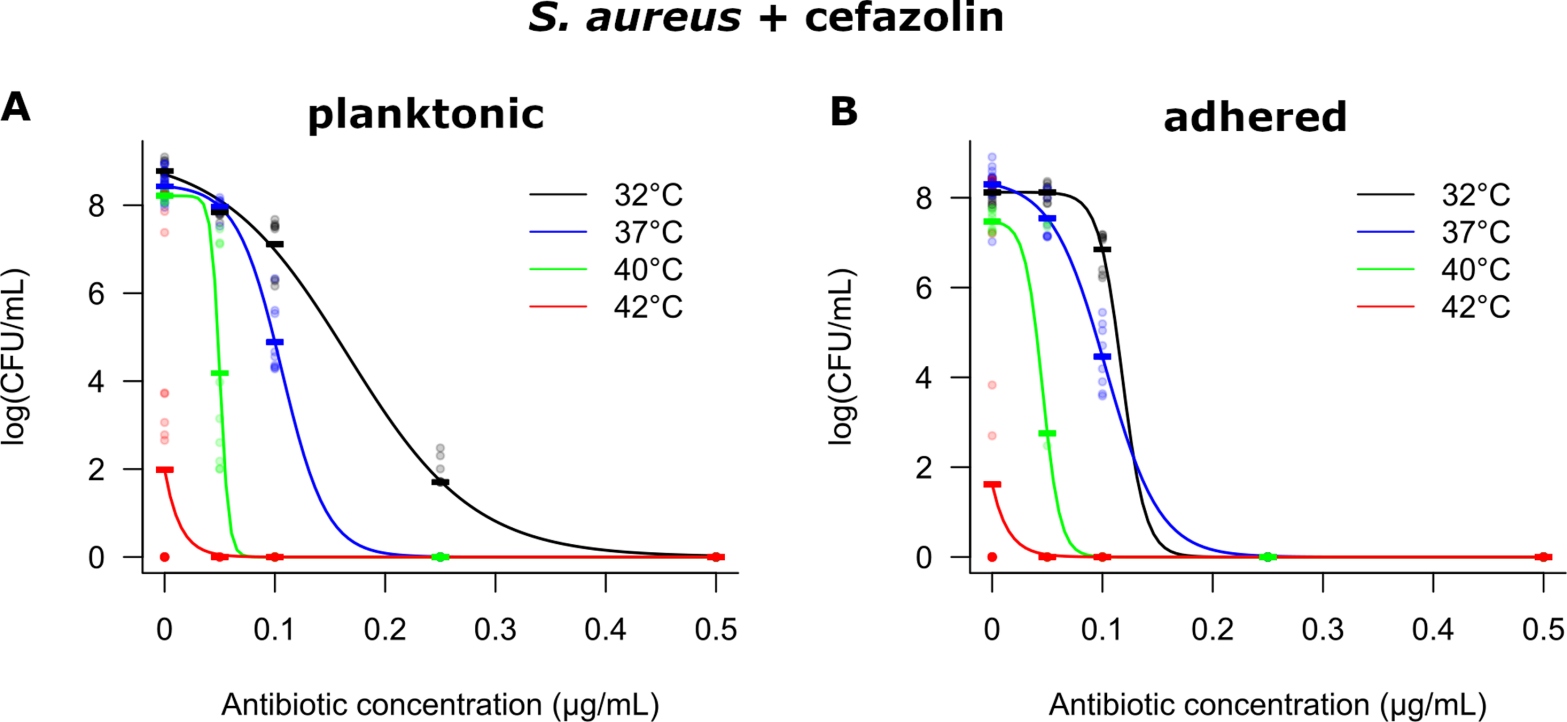
Calculated log-logistic curves for the planktonic **(A)** and adherent **(B)** fractions of *S. aureus* with cefazolin.

Increasing culture temperatures to 42 °C resulted in a decrease in the bacterial load and an increase in susceptibility to gentamicin and cefazolin of both planktonic and adhered *S. aureus* (Figures 1 and 2). Coincidentally, the antibiotic concentration required to kill all bacteria at 42 °C decreased tenfold from 0.5 μg/mL at 32 °C to 0.05 μg/mL for both antibiotics. However, the efficacy of both antibiotics was reduced in presence of 32 °C compared to 37 °C. There were no major differences between the planktonic and adherent groups. For the groups without antibiotics, complete eradication was observed in 18 out of 24 experiments performed at 42 °C, indicating a lack of tolerance of *S. aureus* to these conditions. Calculated log-logistic curves and corresponding ED50 values are reported in Figures 3 and 4 and in Table 1. The obtained ED50 values decreased with increasing temperature, confirming the observed trends from log(CFU/mL) data.

**Table 1:** ED50 values found for different combinations of microorganism and temperature.

Microorganism	Planktonic/Adhered	ED50 32 °C	ED50 37 °C	ED50 40 °C	ED50 42 °C
*S. aureus* (gentamicin)	Planktonic	0.24	0.16	–	N/A
	Adhered	0.25	0.22	–	N/A
*S. aureus* (cefazolin)	Planktonic	0.17	0.11	0.05	N/A
	Adhered	0.12	0.10	0.05	N/A
*S. epidermidis* (cefazolin)	Planktonic	0.14	0.13	0.13	0.09
	Adhered	0.19	0.19	0.16	0.09
*E. coli* (cefazolin)	Planktonic	0.58	0.58	0.62	0.61
	Adhered	0.59	0.57	0.64	0.62

Concentrations are in μg/mL. Fields indicated as N/A represent conditions where the calculated ED50 was <0 μg/mL.

**Figure 5:**
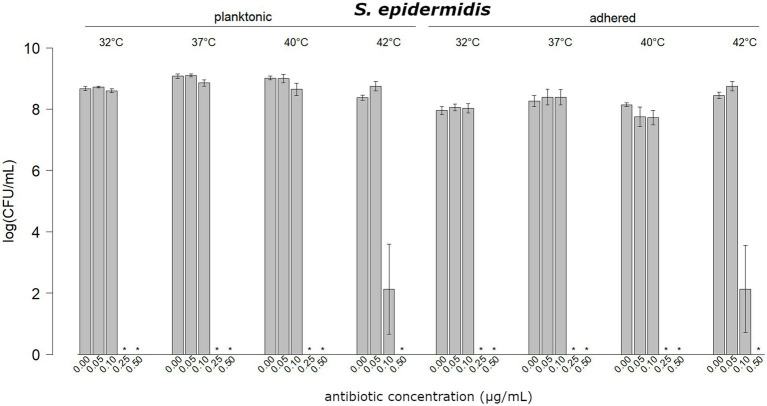
Remaining bacteria titres for *S. epidermidis* with cefazolin after 24 h of culture at different antibiotic concentrations and ambient temperatures. Concentrations are in μg/mL. Results are reported as log(CFU/mL) ± 95% CI.

**Figure 6:**
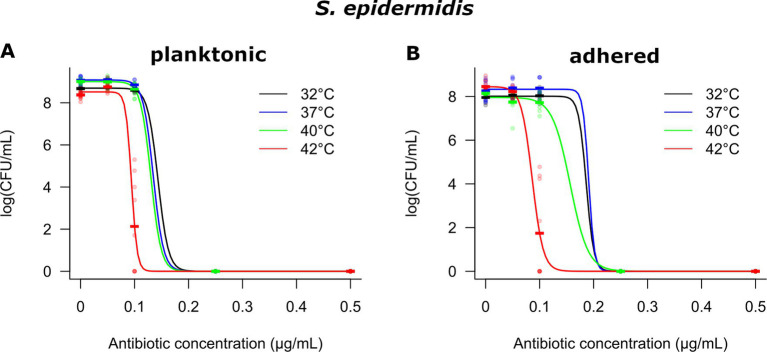
Calculated log-logistic curves for the planktonic **(A)** and adherent **(B)** fractions of *S. epidermidis* with cefazolin.

Compared to *S. aureus,* the effect of temperature on survival and antibiotics susceptibility was less pronounced on *S. epidermidis* (Figure 5). In groups without added antibiotics, no relevant differences were observed in the number of bacteria upon exposure to temperatures ranging from 32 to 40 °C. However, *S. epidermidis* displayed an increased susceptibility to cefazolin at 42 °C compared to other temperature groups, resulting in a 6-log reduction in CFU/m at 0.1 μg/mL of cefazolin. Exposure to these temperatures and/or cefazolin did not cause major differences between planktonic and adhered bacteria. Calculated log-logistic curves and corresponding ED50 values are reported in Figure 6 and Table 1.

**Figure 7:**
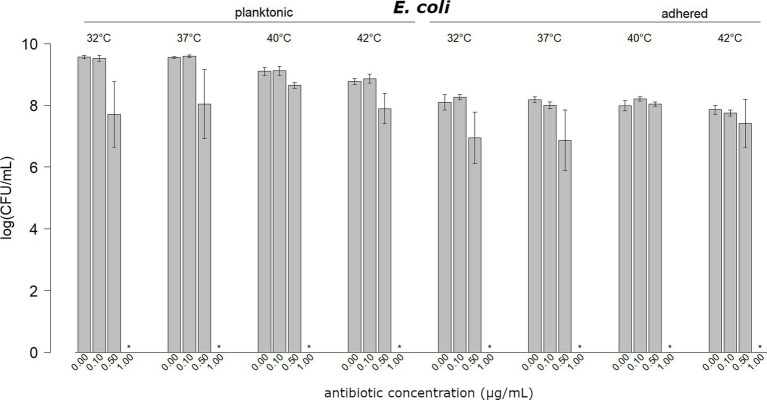
Remaining bacteria titres for *E. coli* with cefazolin after 24 h of culture at different antibiotic concentrations and ambient temperatures. Concentrations are in μg/mL. Results are reported as log(CFU/mL) ± 95% CI.

**Figure 8:**
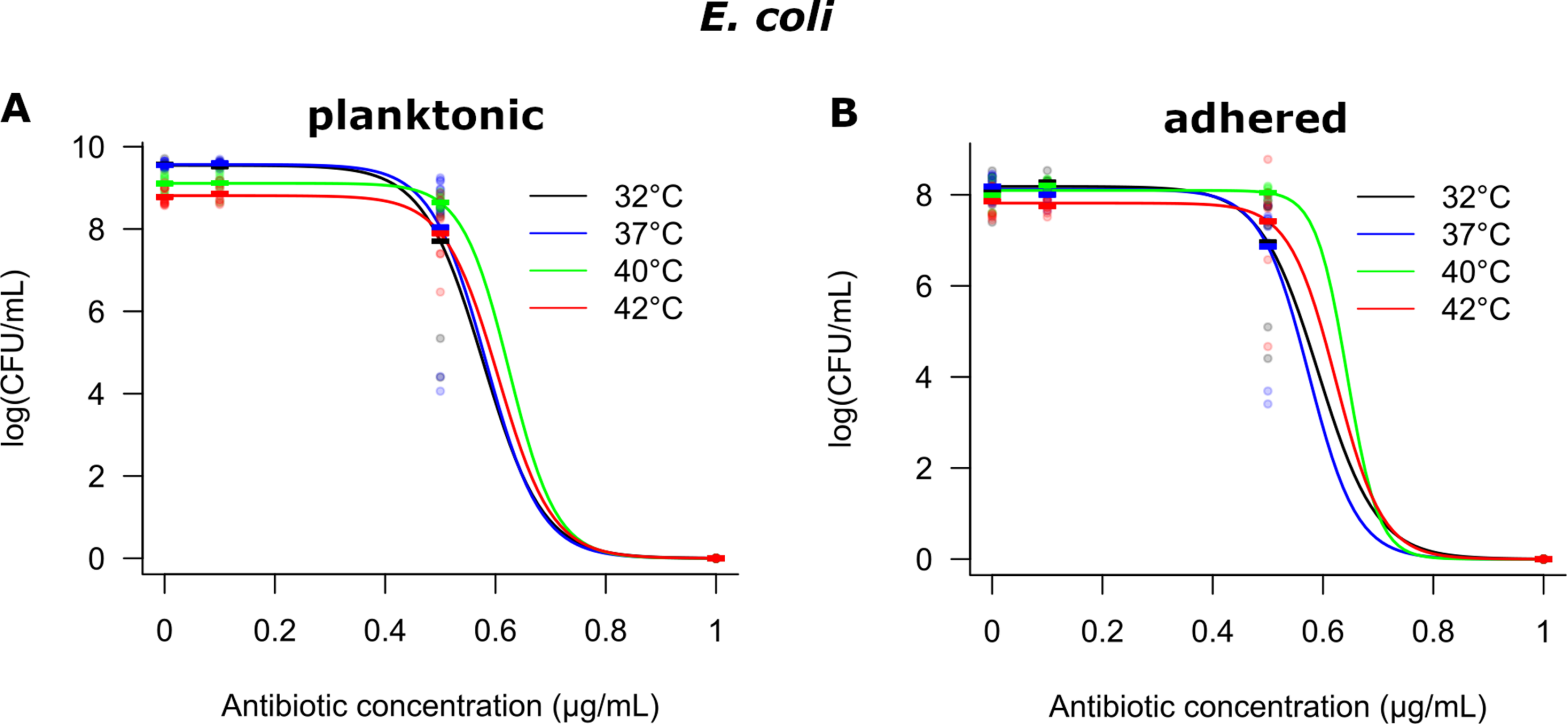
Calculated log-logistic curves for the planktonic **(A)** and adherent **(B)** fractions of *E. coli* with cefazolin.

For *E. coli*, generally higher concentrations of cefazolin were required for a relevant reduction in bacterial load compared to the other bacterial strains. Furthermore, the culturing temperature did not influence the antibiotic susceptibility of *E. coli* (Figure 7). Although a slight reduction in the number of planktonic bacteria was observed upon exposure to increasing temperatures (from ~3.5 * 10^9^ at 32 °C to ~6.5 * 10^8^ at 42 °C), these differences are not considered clinically significant, as it would be unlikely to change patient outcomes. There were no major differences in the response to temperature or antibiotics between planktonic and adhered *E. coli*. Calculated log-logistic curves and corresponding ED50 values are reported in Figure 8 and Table 1.

## 4 Discussion

### 4.1 General findings

This study evaluated the effect of temperature on the killing, surface adhesion and antibiotic susceptibility of pathogenic bacteria. The observed impact of the temperature gradient from 32 to 42 °C differed for each bacterial species. For *S. aureus*, there was a trend of increased susceptibility to antibiotics with higher temperature, and even without antibiotics, eradication of *S. aureus* occurred in almost all replicates at 42 °C. For *S. epidermidis*, an increase in the susceptibility to cefazolin was observed at 42 °C compared to lower temperatures, although an increase temperature alone was not able to reduce the number of bacteria. The gram-negative *E. coli* responded minimally to changes in temperature. *E. coli* is known as a generalist bacterium capable of surviving both in and outside of the body, which could explain its tolerance to temperature changes (Lenski and Benett, 1993; Elsas *et al.* Van Elsas et al., 2011). The effect of temperature on bacterial killing and antibiotic susceptibility is likely caused by combined stress from thermal injury and damage caused by the antibiotics. Heat shock in bacteria leads to damage to membrane proteins, resulting in reduced respiration capacity and a loss of membrane polarisation (Kramer and Thielmann, 2016). At the same time, cefazolin and gentamicin prevent repairs to the cell wall by inhibiting peptidoglycan crosslinking or ribosomal function, respectively (Hahn and Sarre, 1969; Yotsuji et al., 1988). This combination of stress factors could lead to the synergistic effect observed in *S. aureus* and *S. epidermidis*.

The outcomes of our *in vitro* experiments suggest that mild hyperthermia could play an important role in the prevention of PJI, of which *S. aureus* and *S. epidermidis* are the primary causative organisms (Benito et al., 2016; Triffault-Fillit et al., 2019). Furthermore, antibiotic prophylaxis could more effective under normothermia, compared to hypothermic conditions. The joints of patients undergoing total joint arthroplasty are often hypothermic during or after surgery, due to the conditions in the operating room (cool air, continuous air flow and exposed skin) (Kurz et al., 1996; Simpson et al., 2018). Additionally, distal joints such as knees and ankles are naturally cooler than the core, especially when blood perfusion is limited during (i.e., use of tourniquet) and directly after surgery (due to hematoma formation) (Hupel et al., 2000; Gavish et al., 2023; De Marziani et al., 2025). Thus, a simple intraoperative modality like the use of hyperthermia as an adjunct to antibiotic prophylaxis during arthroplasty surgery could be explored in future research. Combined with our findings, it is possible that maintaining a temperature of 37 °C could help prevent *S. aureus* infections. Moreover, lower body temperature has been shown to delay wound healing and suppresses the native immune response (Kurz et al., 1996). The benefits of normothermia on infection prevention could therefore extend to other causative microorganisms, although we have no data to prove or disprove this hypothesis currently.

Our findings also carry implications for other *in vitro* microbiology studies. The vast majority of microbiology experiments are performed at 37 °C, or 35 °C if certain ISO protocols are followed, such as ISO 20776-1 to determine MICs for specific antibiotic-microorganism pairings (ISO 22196 - Measurement of antibacterial activity on plastics and other non-porous surfaces, 2011). However, as discussed here, temperatures can be lower (in distal parts of the body or in case of hypothermia) or higher (in case of inflammation or fever), potentially resulting in different outcomes. Thus, temperature is an important confounder to be taken into consideration when comparing studies or when translating *in vitro* results to the clinic.

Although our study shows the effect of higher temperatures on bacterial killing, heat delivery mechanisms are not explored here. There are several modalities to achieve hyperthermia in bone such as high-intensity focussed ultrasound (HIFU), radio frequency or microwave heating, non-contact induction heating (NCIH) or perfusion with or immersion in warm water (Behrouzkia et al., 2016; Frazier et al., 2016; Chi et al., 2017; Pijls et al., 2020, 2022; Verheul et al., 2025). For prevention of PJI or fracture related infection, NCIH holds particular promise as it can be used non-invasively to heat intramedullary segments of the implant, and heats only the metal (Pijls et al., 2018; Chopra et al., 2017). Since this study investigates the effect of temperature independently of the delivery mechanism, our results are expected to be applicable irrespective of the chosen technique.

### 4.2 Limitations and strengths

Our study provides a proof of principle for the use of mild hyperthermia as an adjunct modality next to antibiotic prophylaxis in the prevention of PJI. Since the required temperatures are still within the physiological range, the risks for patients are minimal for this type of intervention. Nevertheless, some limitations have to be considered. Firstly, the cefazolin concentrations required to eradicate all bacteria in this study (up to 1μg/mL) are quite low compared to those observed in synovial fluid of patients (up to 200μg/mL) (Thabit et al., 2019). Our results would suggest that PJI caused by non-resistant bacteria could not occur regardless of the temperature, which is not the clinical reality. However, these data do not account for factors that could make the eradication of infection more challenging, such as mistimed prophylaxis, decreased blood perfusion at the surgical site, presence of biofilm or AMR (Hupel et al., 2000; Stefánsdóttir et al., 2009). Lower required doses *in vitro* are therefore not surprising.

Secondly, our model differs from the complex clinical setting in some ways (e.g., cell culture media versus body fluid, polystyrene wells versus tissues and biomaterials). Most notably, the effect of the temperature on the native immune system was not modelled. Generally, higher temperature have a stimulating effect on the human immune system, which is thought to be one of the reasons for natural fever (Evans et al., 2015). As such, mild hypothermia could have the added benefit of increasing the host defence against infection, which was not modelled here.

## 5 Conclusion

The effect of elevated physiological temperatures on bacterial load, adherence and antibiotic susceptibility was investigated in an *in vitro* infection model. The effect of temperature on bacterial survival differed greatly between different microorganisms. *S. aureus* was most affected by elevated temperatures, with significant reduction in bacterial load and increased antibiotic susceptibility at 42 °C. For the other bacterial species tested, the effect of temperature was less pronounced.

## Data Availability

The original contributions presented in the study are included in the article/Supplementary material, further inquiries can be directed to the corresponding author.
