# Supplementary material for: The effect of elevated physiological temperatures on bacterial survival and antibiotic susceptibility in an in vitro infection prevention model
Source: Front Microbiol. 2026 May 29;17:1830056. doi: 10.3389/fmicb.2026.1830056 (PMC13260641; doi:10.3389/fmicb.2026.1830056)
Supplement: Supplementary file 1 [file Table_1.docx]

Supplementary Material

# Supplementary Data – R code

#install.packages("drc")

library(drc)

library(ggplot2)

library(dplyr)

citation("drc")

setwd("R scripts/fever study")

dat1=read.csv("fever data v4.csv",sep=";",dec = ".")

dat2=read.csv("data for barplots.csv",sep=";",dec = ".")

dat1$colours=cut(dat1$temperature, breaks = c(31,33,38,41,43),labels=c("black","blue","green","red"))

dat1$means=0

dat1$SE=0

mean(c(1,2))

for (i in (1:length(dat1$year))){

if(dat1$antibiotic_concentration[i]==0){

tempdat = log(filter(dat1,planktonic==dat1$planktonic[i]&temperature==dat1$temperature[i]&bacteria==dat1$bacteria[i]&antibiotic_concentration==dat1$antibiotic_concentration[i])$CFU.mL+1,10)

dat1[i,13]=mean(tempdat)

dat1[i,14]=sd(tempdat)/sqrt(length(tempdat))

}else{

tempdat=log(filter(dat1,planktonic==dat1$planktonic[i]&temperature==dat1$temperature[i]&bacteria==dat1$bacteria[i]&antibiotic==dat1$antibiotic[i]&antibiotic_concentration==dat1$antibiotic_concentration[i])$CFU.mL+1,10)

dat1[i,13]=mean(tempdat)

dat1[i,14]=sd(tempdat)/sqrt(length(tempdat))

}

}

#converts temperatures from numberical to strings with degree sign

for (i in (1:length(dat1$temperature))){

dat1[i,6]=paste(toString(dat1[i,6]),"°C",sep="")

}

###log curves#####

#aureus genta

dat=filter(dat1, bacteria=="S. aureus" & planktonic==1&antibiotic=="genta"|(bacteria=="S. aureus" & planktonic==1&antibiotic=="cefzol"&antibiotic_concentration==0&temperature!="40°C"))

test = drm(log(CFU.mL+1,10)~antibiotic_concentration, temperature, data=dat,logDose=10,fct=LL.3(names=c("slope","Upper Limit","ED50")))

plot(test, main ="S. aureus planktonic + gentamicin",col=c("black","blue","red"),pch="",lwd=1.5,lty=1,bty="l",xlab="Antibiotic concentration (μg/mL)",ylab="log(CFU/mL)")

points(dat$antibiotic_concentration,log(dat$CFU.mL+1,10), pch=20, cex=0.8, col=alpha(dat$colours, 0.2))

points(dat$antibiotic_concentration,dat$means, pch="-", cex=2, col=alpha(dat$colours, 1))

summary(test)

log(coef(test)[7:9],10)

dat=filter(dat1, bacteria=="S. aureus" & planktonic==0&antibiotic=="genta"|(bacteria=="S. aureus" & planktonic==0&antibiotic=="cefzol"&antibiotic_concentration==0&temperature!="40°C"))

test = drm(log(CFU.mL+1,10)~antibiotic_concentration, temperature, data=dat,logDose=10,fct=LL.3(names=c("slope","Upper Limit","ED50")))

plot(test, main ="S. aureus adherent + gentamicin",col=c("black","blue","red"),pch="",lwd=1.5,lty=1,bty="l",xlab="Antibiotic concentration (μg/mL)",ylab="log(CFU/mL)")

points(dat$antibiotic_concentration,log(dat$CFU.mL+1,10), pch=20, cex=0.8, col=alpha(dat$colours, 0.2))

points(dat$antibiotic_concentration,dat$means, pch="-", cex=2, col=alpha(dat$colours, 1))

summary(test)

log(coef(test)[7:9],10)

#aureus cefzol

dat=filter(dat1, bacteria=="S. aureus" & planktonic==1&antibiotic=="cefzol"|(bacteria=="S. aureus" & planktonic==1&antibiotic=="genta"&antibiotic_concentration==0))

test = drm(log(CFU.mL+1,10)~antibiotic_concentration, temperature, data=dat,logDose=10,fct=LL.3(names=c("slope","Upper Limit","ED50")))

plot(test, main ="S. aureus planktonic + cefazolin",col=c("black","blue","green","red"),pch="",lwd=1.5,lty=1,bty="l",xlab="Antibiotic concentration (μg/mL)",ylab="log(CFU/mL)")

points(dat$antibiotic_concentration,log(dat$CFU.mL+1,10), pch=20, cex=0.8, col=alpha(dat$colours, 0.2))

points(dat$antibiotic_concentration,dat$means, pch="-", cex=2, col=alpha(dat$colours, 1))

summary(test)

log(coef(test)[9:12],10)

dat=filter(dat1, bacteria=="S. aureus" & planktonic==0&antibiotic=="cefzol"|(bacteria=="S. aureus" & planktonic==0&antibiotic=="genta"&antibiotic_concentration==0))

test = drm(log(CFU.mL+1,10)~antibiotic_concentration, temperature, data=dat,logDose=10,fct=LL.3(names=c("slope","Upper Limit","ED50")))

plot(test, main ="S. aureus adherent + cefazolin",col=c("black","blue","green","red"),pch="",lwd=1.5,lty=1,bty="l",xlab="Antibiotic concentration (μg/mL)",ylab="log(CFU/mL)")

points(dat$antibiotic_concentration,log(dat$CFU.mL+1,10), pch=20, cex=0.8, col=alpha(dat$colours, 0.2))

points(dat$antibiotic_concentration,dat$means, pch="-", cex=2, col=alpha(dat$colours, 1))

summary(test)

log(coef(test)[9:12],10)

#epidermidis

dat=filter(dat1, bacteria=="S. epidermidis" & planktonic==1&antibiotic=="cefzol")

test = drm(log(CFU.mL+1,10)~antibiotic_concentration, temperature, data=dat,logDose=10,fct=LL.3(names=c("slope","Upper Limit","ED50")))

plot(test, main ="S. epidermidis planktonic",col=c("black","blue","green","red"),pch="",lwd=1.5,lty=1,bty="l",xlab="Antibiotic concentration (μg/mL)",ylab="log(CFU/mL)")

points(dat$antibiotic_concentration,log(dat$CFU.mL+1,10), pch=20, cex=0.8, col=alpha(dat$colours, 0.2))

points(dat$antibiotic_concentration,dat$means, pch="-", cex=2, col=alpha(dat$colours, 1))

summary(test)

log(coef(test)[9:12],10)

dat=filter(dat1, bacteria=="S. epidermidis" & planktonic==0&antibiotic=="cefzol")

test = drm(log(CFU.mL+1,10)~antibiotic_concentration, temperature, data=dat,logDose=10,fct=LL.3(names=c("slope","Upper Limit","ED50")))

plot(test, main ="S. epidermidis adhered",col=c("black","blue","green","red"),pch="",lwd=1.5,lty=1,bty="l",xlab="Antibiotic concentration (μg/mL)",ylab="log(CFU/mL)")

points(dat$antibiotic_concentration,log(dat$CFU.mL+1,10), pch=20, cex=0.8, col=alpha(dat$colours, 0.2))

points(dat$antibiotic_concentration,dat$means, pch="-", cex=2, col=alpha(dat$colours, 1))

summary(test)

log(coef(test)[9:12],10)

#coli

dat=filter(dat1, bacteria=="E. coli" & planktonic==1&antibiotic=="cefzol")

test = drm(log(CFU.mL+1,10)~antibiotic_concentration, temperature, data=dat,logDose=10,fct=LL.3(names=c("slope","Upper Limit","ED50")))

plot(test, main ="E. coli planktonic",col=c("black","blue","green","red"),pch="",lwd=1.5,lty=1,bty="l",xlab="Antibiotic concentration (μg/mL)",ylab="log(CFU/mL)")

points(dat$antibiotic_concentration,log(dat$CFU.mL+1,10), pch=20, cex=0.8, col=alpha(dat$colours, 0.2))

points(dat$antibiotic_concentration,dat$means, pch="-", cex=2, col=alpha(dat$colours, 1))

log(coef(test)[9:12],10)

dat=filter(dat1, bacteria=="E. coli" & planktonic==0&antibiotic=="cefzol")

test = drm(log(CFU.mL+1,10)~antibiotic_concentration, temperature, data=dat,logDose=10,fct=LL.3(names=c("slope","Upper Limit","ED50")))

plot(test, main ="E. coli adhered",col=c("black","blue","green","red"),pch="",lwd=1.5,lty=1,bty="l",xlab="Antibiotic concentration (μg/mL)",ylab="log(CFU/mL)")

points(dat$antibiotic_concentration,log(dat$CFU.mL+1,10), pch=20, cex=0.8, col=alpha(dat$colours, 0.2))

points(dat$antibiotic_concentration,dat$means, pch="-", cex=2, col=alpha(dat$colours, 1))

summary(test)

log(coef(test)[9:12],10)

####

#bargraphs

#####

#aureus genta

dat2

dat=filter(dat2,bacteria=="aureus_genta")

subdat=filter(dat,planktonic==1)

subdat2=filter(dat,planktonic==0)

subdat

SEs = as.numeric(c(

subdat[1,11:16],0.0001,0.0001,

subdat[2,11:16],0.0001,0.0001,

subdat[3,11:16],0.0001,0.0001,

subdat2[1,11:16],0.0001,0.0001,

subdat2[2,11:16],0.0001,0.0001,

subdat2[3,11:16]

))

heights= as.numeric(c(

subdat[1,4:9],0.0001,0.0001,

subdat[2,4:9],0.0001,0.0001,

subdat[3,4:9],0.0001,0.0001,

subdat2[1,4:9],0.0001,0.0001,

subdat2[2,4:9],0.0001,0.0001,

subdat2[3,4:9]

))

names=c(

"0.00","0.01","0.05","0.10","0.25","0.50","","",

"0.00","0.01","0.05","0.10","0.25","0.50","","",

"0.00","0.01","0.05","0.10","0.25","0.50","","",

"0.00","0.01","0.05","0.10","0.25","0.50","","",

"0.00","0.01","0.05","0.10","0.25","0.50","","",

"0.00","0.01","0.05","0.10","0.25","0.50"

)

tempnames=c("32°C","37°C","42°C")

bar=barplot(heights,xlab="antibiotic concentration (μg/mL)",ylab="log(CFU/mL)",las=2,ylim=c(0,10),cex.axis=1.5,cex.lab=1.4)

lines(x=c(0,bar[length(bar)]),y=c(0,0))

lines(x=c(bar[1],bar[length(bar)/2]),y=c(10.7,10.7),xpd=TRUE)

lines(x=c(bar[length(bar)/2+1],bar[length(bar)]),y=c(10.7,10.7),xpd=TRUE)

arrows(x0=bar,y0=heights-SEs*1.96,y1=heights+SEs*1.96,angle=90,code=3,length=0.03,xpd=TRUE)

text(x=bar-0.45,y=-0.35,names,srt=45,xpd=TRUE, cex=0.95)

text(x=c(bar[length(bar)/6-3],bar[length(bar)/6*2-3],bar[length(bar)/6*3-3],bar[length(bar)/6*4-3],bar[length(bar)/6*5-3],bar[length(bar)-3]),y=10.2,tempnames,xpd=TRUE,cex=1.1)

text(x=c(bar[length(bar)/4],bar[length(bar)/4*3]),y=11.1,c("planktonic","adhered"),xpd=TRUE,cex=1.3)

text(x=bar[length(bar)/2],y=11.2,"S. aureus + gentamicin",xpd=TRUE,cex=1.8)

for (i in 1:length(bar)){

if (heights[i]==0){

text(x=bar[i],y=0.2,"*")

}

}

#aureus cefzol

dat=filter(dat2,bacteria=="aureus_cefzol")

subdat=filter(dat,planktonic==1)

subdat2=filter(dat,planktonic==0)

subdat2

SEs = as.numeric(c(

subdat[1,11],subdat[1,13:16],0,0,

subdat[2,11],subdat[2,13:16],0,0,

subdat[3,11],subdat[3,13:16],0,0,

subdat[4,11],subdat[4,13:16],0,0,

subdat2[1,11],subdat2[1,13:16],0,0,

subdat2[2,11],subdat2[2,13:16],0,0,

subdat2[3,11],subdat2[3,13:16],0,0,

subdat2[4,11],subdat2[4,13:16]

))

heights= as.numeric(c(

subdat[1,4],subdat[1,6:9],0.0001,0.0001,

subdat[2,4],subdat[2,6:9],0.0001,0.0001,

subdat[3,4],subdat[3,6:9],0.0001,0.0001,

subdat[4,4],subdat[4,6:9],0.0001,0.0001,

subdat2[1,4],subdat2[1,6:9],0.0001,0.0001,

subdat2[2,4],subdat2[2,6:9],0.0001,0.0001,

subdat2[3,4],subdat2[3,6:9],0.0001,0.0001,

subdat2[4,4],subdat2[4,6:9]

))

names=c(

"0.00","0.05","0.10","0.25","0.50","","",

"0.00","0.05","0.10","0.25","0.50","","",

"0.00","0.05","0.10","0.25","0.50","","",

"0.00","0.05","0.10","0.25","0.50","","",

"0.00","0.05","0.10","0.25","0.50","","",

"0.00","0.05","0.10","0.25","0.50","","",

"0.00","0.05","0.10","0.25","0.50","","",

"0.00","0.05","0.10","0.25","0.50"

)

tempnames=c("32°C","37°C","40°C","42°C")

bar=barplot(heights,xlab="antibiotic concentration (μg/mL)",ylab="log(CFU/mL)",las=2,ylim=c(0,10),cex.axis=1.5,cex.lab=1.4)

lines(x=c(0,bar[length(bar)]),y=c(0,0))

lines(x=c(bar[1],bar[length(bar)/2]),y=c(10.7,10.7),xpd=TRUE)

lines(x=c(bar[length(bar)/2+1],bar[length(bar)]),y=c(10.7,10.7),xpd=TRUE)

arrows(x0=bar,y0=heights-SEs*1.96,y1=heights+SEs*1.96,angle=90,code=3,length=0.03,xpd=TRUE)

text(x=bar-0.4,y=-0.3,names,srt=45,xpd=TRUE, cex=0.95)

text(x=c(bar[length(bar)/8-3],bar[length(bar)/8*2-3],bar[length(bar)/8*3-3],bar[length(bar)/8*4-3],bar[length(bar)/8*5-3],bar[length(bar)/8*6-3],bar[length(bar)/8*7-3],bar[length(bar)/8*8-3]),y=10.2,tempnames,xpd=TRUE,cex=1.1)

text(x=c(bar[13],bar[41]),y=11,c("planktonic","adhered"),xpd=TRUE,cex=1.3)

text(x=bar[length(bar)/2],y=11.2,"S. aureus + cefazolin",xpd=TRUE,cex=1.8)

for (i in 1:length(bar)){

if (heights[i]==0){

text(x=bar[i],y=0.2,"*")

}

}

#epidermidis cefzol

dat=filter(dat2,bacteria=="epidermidis")

subdat=filter(dat,planktonic==1)

subdat2=filter(dat,planktonic==0)

subdat

SEs = as.numeric(c(

subdat[1,11],subdat[1,13:16],0,0,

subdat[2,11],subdat[2,13:16],0,0,

subdat[3,11],subdat[3,13:16],0,0,

subdat[4,11],subdat[4,13:14],subdat[4,16],0,0,

subdat2[1,11],subdat2[1,13:16],0,0,

subdat2[2,11],subdat2[2,13:16],0,0,

subdat2[3,11],subdat2[3,13:16],0,0,

subdat2[4,11],subdat2[4,13:14],subdat2[4,16]

))

subdat

heights= as.numeric(c(

subdat[1,4],subdat[1,6:9],0.0001,0.0001,

subdat[2,4],subdat[2,6:9],0.0001,0.0001,

subdat[3,4],subdat[3,6:9],0.0001,0.0001,

subdat[4,4],subdat[4,6:7],subdat[4,9],0.0001,0.0001,

subdat2[1,4],subdat2[1,6:9],0.0001,0.0001,

subdat2[2,4],subdat2[2,6:9],0.0001,0.0001,

subdat2[3,4],subdat2[3,6:9],0.0001,0.0001,

subdat2[4,4],subdat[4,6:7],subdat[4,9]

))

names=c(

"0.00","0.05","0.10","0.25","0.50","","",

"0.00","0.05","0.10","0.25","0.50","","",

"0.00","0.05","0.10","0.25","0.50","","",

"0.00","0.05","0.10","0.50","","",

"0.00","0.05","0.10","0.25","0.50","","",

"0.00","0.05","0.10","0.25","0.50","","",

"0.00","0.05","0.10","0.25","0.50","","",

"0.00","0.05","0.10","0.50"

)

tempnames=c("32°C","37°C","40°C","42°C")

bar=barplot(heights,xlab="antibiotic concentration (μg/mL)",ylab="log(CFU/mL)",las=2,ylim=c(0,10),cex.axis=1.5,cex.lab=1.4)

lines(x=c(0,bar[length(bar)]),y=c(0,0))

lines(x=c(bar[1],bar[length(bar)/2]),y=c(10.7,10.7),xpd=TRUE)

lines(x=c(bar[length(bar)/2+1],bar[length(bar)]),y=c(10.7,10.7),xpd=TRUE)

arrows(x0=bar,y0=heights-SEs*1.96,y1=heights+SEs*1.96,angle=90,code=3,length=0.03,xpd=TRUE)

text(x=bar-0.4,y=-0.3,names,srt=45,xpd=TRUE, cex=0.95)

text(x=c(bar[3],bar[10],bar[17],bar[24],bar[30],bar[37],bar[44],bar[51]),y=10.2,tempnames,xpd=TRUE,cex=1.1)

text(x=c(bar[13],bar[41]),y=11,c("planktonic","adhered"),xpd=TRUE,cex=1.3)

text(x=bar[length(bar)/2],y=11.2,"S. epidermidis + cefazolin",xpd=TRUE,cex=1.8)

for (i in 1:length(bar)){

if (heights[i]==0){

text(x=bar[i],y=0.2,"*")

}

}

#coli cefzol

dat=filter(dat2,bacteria=="coli")

subdat=filter(dat,planktonic==1)

subdat2=filter(dat,planktonic==0)

SEs = as.numeric(c(

subdat[1,11],subdat[1,14],subdat[1,16:17],0.0001,0.0001,

subdat[2,11],subdat[2,14],subdat[2,16:17],0.0001,0.0001,

subdat[3,11],subdat[3,14],subdat[3,16:17],0.0001,0.0001,

subdat[4,11],subdat[4,14],subdat[4,16:17],0.0001,0.0001,

subdat2[1,11],subdat2[1,14],subdat2[1,16:17],0.0001,0.0001,

subdat2[2,11],subdat2[2,14],subdat2[2,16:17],0.0001,0.0001,

subdat2[3,11],subdat2[3,14],subdat2[3,16:17],0.0001,0.0001,

subdat2[4,11],subdat2[4,14],subdat2[4,16:17]

))

heights= as.numeric(c(

subdat[1,4],subdat[1,7],subdat[1,9:10],0.0001,0.0001,

subdat[2,4],subdat[2,7],subdat[2,9:10],0.0001,0.0001,

subdat[3,4],subdat[3,7],subdat[3,9:10],0.0001,0.0001,

subdat[4,4],subdat[4,7],subdat[4,9:10],0.0001,0.0001,

subdat2[1,4],subdat2[1,7],subdat2[1,9:10],0.0001,0.0001,

subdat2[2,4],subdat2[2,7],subdat2[2,9:10],0.0001,0.0001,

subdat2[3,4],subdat2[3,7],subdat2[3,9:10],0.0001,0.0001,

subdat2[4,4],subdat2[4,7],subdat2[4,9:10]

))

names=c(

"0.00","0.10","0.50","1.00","","",

"0.00","0.10","0.50","1.00","","",

"0.00","0.10","0.50","1.00","","",

"0.00","0.10","0.50","1.00","","",

"0.00","0.10","0.50","1.00","","",

"0.00","0.10","0.50","1.00","","",

"0.00","0.10","0.50","1.00","","",

"0.00","0.10","0.50","1.00"

)

tempnames=c("32°C","37°C","40°C","42°C")

bar=barplot(heights,xlab="antibiotic concentration (μg/mL)",ylab="log(CFU/mL)",las=2,ylim=c(0,10),cex.axis=1.5,cex.lab=1.4)

lines(x=c(0,bar[length(bar)]),y=c(0,0))

lines(x=c(bar[1],bar[length(bar)/2]),y=c(10.7,10.7),xpd=TRUE)

lines(x=c(bar[length(bar)/2+1],bar[length(bar)]),y=c(10.7,10.7),xpd=TRUE)

arrows(x0=bar,y0=heights-SEs*1.96,y1=heights+SEs*1.96,angle=90,code=3,length=0.03,xpd=TRUE)

text(x=bar-0.4,y=-0.3,names,srt=45,xpd=TRUE, cex=0.95)

text(x=c(bar[length(bar)/8-3],bar[length(bar)/8*2-3],bar[length(bar)/8*3-3],bar[length(bar)/8*4-3],bar[length(bar)/8*5-2],bar[length(bar)/8*6-2],bar[length(bar)/8*7-2],bar[length(bar)/8*8-2]),y=10.2,tempnames,xpd=TRUE,cex=1.1)

text(x=c(bar[13],bar[41]),y=11,c("planktonic","adhered"),xpd=TRUE,cex=1.3)

text(x=bar[length(bar)/2],y=11.2,"E. coli + cefazolin",xpd=TRUE,cex=1.8)

for (i in 1:length(bar)){

if (heights[i]==0){

text(x=bar[i],y=0.2,"*")

}

}
